# Comparing Isolated Thumb Force Generation, Wrist Rotation, and Clinical Measurements in Healthy and Osteoarthritic Individuals before and after Surgery

**DOI:** 10.3390/bioengineering11090948

**Published:** 2024-09-22

**Authors:** Nicole Arnold, Adam Chrzan, Kevin Chan, Daniel Hess, Levi Hinkleman, Stephen Duquette, John Kelpin, Tamara Reid Bush

**Affiliations:** 1 Mechanical Engineering, Michigan State University, East Lansing, MI 48824, USA; arnoldn5@msu.edu (N.A.); chrzanad@msu.edu (A.C.); 2 Orthopedic Hand and Upper Extremity Surgery, Corewell Health West, Grand Rapids, MI 49546, USA; chank85@gmail.com (K.C.); daniel.hess@corewellhealth.org (D.H.); levi.hinkelman@corewellhealth.org (L.H.); stephen.duquette@corewellhealth.org (S.D.); john.kelpin@corewellhealth.org (J.K.)

**Keywords:** osteoarthritis, surgery, kinetics, thumb

## Abstract

Thumb carpometacarpal (CMC) osteoarthritis (OA) is caused by the degeneration of joint surfaces at the base of the thumb. If conservative treatments have failed, surgery may be needed to improve symptoms. Typically, standard clinical tools, such as the pinch gauge, are used to measure thumb force. However, these devices have utilized multiple digits and do not represent forces specifically generated by the thumb. Therefore, different devices are necessary to accurately measure isolated thumb force. The primary objective was to research the effect of thumb force after ligament reconstruction with tendon interposition surgery. To accomplish this, several sub-objectives were implemented: (1) create a testing device to collect isolated thumb forces, (2) collect a normative thumb force data set of males and females to compare the impact of aging and surgery, (3) collect and compare clinical data to see if these data sets matched isolated thumb forces, (4) determine the effect of wrist position on isolated thumb force data in different wrist positions, and (5) collect thumb force in directions that mimic daily activities, a directional force downward (push) and inward (pull). On average, older participants generated statistically larger forces than younger participants. Additionally, only 50% of CMC OA participants showed greater than 5 N of improvement at 6-months post-surgery compared to pre-surgery, but did not reach healthy force levels. When evaluating wrist rotation, OA participants’ push and pull decreased by 8 N and 7 N in the horizontal wrist position, and their push and pull increased by 2 N and 5 N in the vertical wrist position. Evaluation and results with standard clinical tools showed different post-surgery trends than isolated force data, which suggested the clinical approach has mixed results and may be under- or over-estimating the recovery process. These data sets allow surgeons and hand therapists to identify changes in isolated thumb force generation to create specialized therapies and treatment options, which is an improvement upon current clinical measurement tools.

## 1. Introduction

Nearly 76% of the general population has been impacted by hand osteoarthritis (OA) [1]. Hand OA is a debilitating and prevalent disorder caused by the degeneration of joint surfaces and cartilage [2,3]. The carpometacarpal (CMC) joint, located at the base of the thumb, is described as a common location of hand OA [4]. Specific causes of CMC OA have not yet been confirmed; however, multiple studies have reported ligament laxity to be a likely factor [5,6,7]. Ligament laxity has led to joint misalignment and altered loading distribution paths along the thumb [8]. Repetitive joint loading and localized joint damage are also known to be risk factors of thumb CMC OA [9,10,11,12]. Other risk factors for developing hand OA were reported to be age and sex [12,13]. The prevalence of CMC OA has been reported to be 1 out of 12 older men and 1 out of 4 postmenopausal women [2,14,15]. Irrespective of age or sex, individuals with CMC OA may experience joint pain, stiffness, decreased strength, and reduced range of motion of the hand and thumb [16]. These reductions in function lead to the inability to grasp, manipulate, and hold items such as cooking utensils or clothing while dressing oneself [2,17,18]. Overall, the reduction in function from CMC OA also results in reduced productivity, lack of independence, and the inability to care for oneself, thus severely impacting one’s quality of life [19].

In order to improve the quality of life for those living with severe CMC OA, surgical treatment is conducted to relieve pain and restore function [2,20]. Although there are many surgical options, the most common surgical procedure for those with thumb CMC OA is ligament reconstruction with tendon interposition (LRTI) [21,22,23]. The LRTI surgery has been documented to effectively reduce pain in over 70% of patients [5,6,24]. However, thumb force generation has not been well researched among CMC OA patients pre- and post-surgery, specifically at multiple time points post-surgery. Due to this gap in research, there is a need to investigate the effect of LRTI surgery to assess changes in thumb force.

Standard clinical tools, the dynamometer and pinch gauge, are often used to assess thumb force changes before and after LRTI surgery. These tools collect measurements such as grip strength (measured by the dynamometer), tip, and key pinch (measured by the pinch gauge). However, these devices utilize multiple digits to obtain data. Therefore, these clinical devices are not representative of the forces specifically produced only by the thumb. Furthermore, grip strength has been known to be affected by both wrist position and angle [25,26,27,28,29,30,31]. Studies have reported on the effect of wrist position (e.g., rotation, flexed, or extended) on grip strength, but have shown mixed results detailing the strongest position [32,33,34,35,36,37]. Nonetheless, it should again be noted that grip strength does not measure forces produced by only the thumb. To accurately measure isolated thumb force and at various wrist positions, different devices are necessary.

Common daily activities position the thumb in a variety of angles to execute manipulative tasks [34]. Some tasks can require an applied force inward toward the hand (i.e., as in holding a knife), or an applied force downward (i.e., as in typing on a keyboard). However, few studies have researched the impact of isolated thumb forces applied in various directions. Clinical measurement tools aim to measure thumb force in positions and directions that simulate everyday activities (e.g., key pinch or holding and inserting a key into a lock), but these measurements of the thumb force are often masked by the assistance of other digits. Therefore, there is a need to accurately capture isolated thumb forces in the mannerisms of daily activities, i.e., applying force in different directions similar to common tasks, to identify thumb force deficits pre- and post-surgery [34].

There were a limited number of research studies that obtained isolated thumb forces. One study measured thumb force where the palm faced downward and medially. The palm down position produced more thumb force than in the medial position [35]. It is important to note that these studies were limited to a population of only young healthy males, which are not representative of the population diagnosed with CMC OA [31,35]. Other studies tested the effect of an exercise regimen on thumb force in four directions (with the palm oriented medially) and also with a custom force measurement device in healthy (younger and older) and arthritic individuals at three time points [36,37]. Additionally, other studies have utilized custom devices to collect thumb forces. One study measured thumbtip force, but with a small sample size of younger individuals [38,39]. Studies conducted by Crisco et al. developed a custom cylindrical testing device to measure thumb force applied during a jar grasp in those with CMC OA and a control population, which found early thumb CMC OA was associated with weaker cylindrical grasp [40,41]. Thus, further research is needed on the differences in isolated thumb forces in the OA and healthy populations.

Our primary objective was to research the effect of thumb force after LRTI surgery. In order to assess this objective, a few sub-objectives needed to also be considered. The first sub-objective was to create a testing device that would enable the collection of isolated thumb forces. Second, for comparison purposes to identify how much function was lost, it was necessary to obtain and analyze normative thumb force data sets for both males and female controls. These data sets were used as a normative baseline for comparison to individuals with CMC OA. Because the diagnosis of CMC OA is known to increase with age, two age groups were included and compared to better understand the progression of aging on force generation. A third sub-objective was implemented to collect and compare clinical data to the isolated thumb forces obtained with the new device. Research has also shown wrist position affects thumb and hand strength. Additionally, the literature also lacks thumb force data in different directions as they relate to activities of daily living and the impact surgery may have on the ability to execute these activities. The testing device was designed to capture thumb force in a wide variety of thumb postures to capture a more complete picture post-surgery. Thus, the last sub-objectives were to determine the effect of wrist position on isolated thumb force data and how direction impacted force generation. Specifically, directions that mimic daily activities, a directional force downward (push) and inward (pull), were evaluated.

## 2. Materials and Methods

### 2.1. Participant Testing

All testing and participant data were collected in accordance with Michigan State University’s Institutional Review Board and all individuals consented (IRB#00006111, IRB#00006525, IRB#2021-148). Healthy participants were identified as those who did not have diagnosed hand or thumb OA, no prior surgery or injury to their thumb, were right-handed, over the age of 18, and were not pregnant. Two healthy groups of males and females were tested: younger healthy (YH) and older healthy (OH). Younger healthy participants were between the ages of 18 and 39, while older healthy participants were those over the age of 40. An additional test group included surgical participants with doctor-diagnosed thumb CMC OA who had consented to LRTI surgery. Healthy participants were tested once on their right hand, and surgical participants were tested before surgery, at 3 months, and 6 months after surgery on the hand that received surgery. All OA participants had surgery at the same clinic and were prescribed a standard postoperative rehabilitation routine. The CMC joint was immobilized in a splint for 4–6 weeks after surgery.

### 2.2. Equipment for Clinical Strength Measures

**Figure 1:**
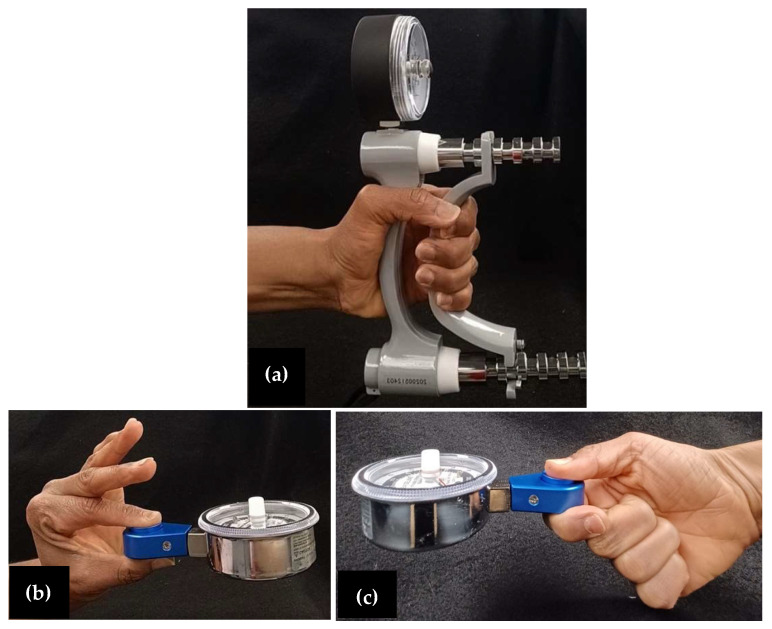
(**a**) The Jamar dynamometer was used to measure hand grip strength. Participants were instructed to use the whole hand to grasp the whole device and squeeze as hard as possible for 2–3 s. The Baseline hydraulic pinch gauge was used to measure tip and key pinch. (**b**) Tip pinch was performed with the index finger superiorly and the thumb inferiorly to execute a “pinch” motion, squeezing the button as strength was recorded. (**c**) Key pinch was performed with the thumb placed superiorly and with the radial middle aspect of index finger underneath the button to squeeze as strength was recorded. Grip and pinch strength measurements were recorded and averaged across three trials.

Clinical measurement testing included grip strength, tip pinch, and key pinch. Grip strength was collected using the Jamar Hydraulic Hand Dynamometer, (Model 081028935, Cedarburg, WI, USA) shown in Figure 1a. Tip (Figure 1b) and key pinch (Figure 1c) were collected using the Baseline hydraulic pinch gauge (Model 12-0235, White Plains, NY, USA). All data reported for the grip and pinch strength are the average of three trials.

### 2.3. Force Equipment and Testing Setup

**Figure 2:**
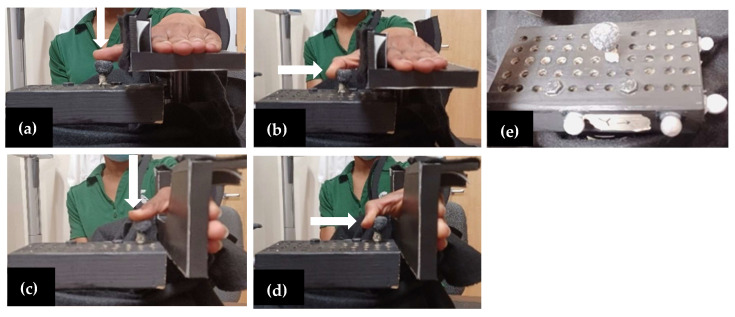
(**a**) 0° wrist position thumb push towards the ground, (**b**) 0° wrist position thumb pull in towards the palm, (**c**) 90° wrist position thumb push towards the ground, (**d**) 90° wrist position thumb pull in towards the palm, (**e**) block system was mounted on the load cell with equally spaced holes where participants placed the peg.

In this study, a custom testing device was created to isolate force generated by the thumb in various postures. The custom testing device included a block mounted on a six-axis load cell (AMTI, Boston, MA, USA), and data were collected at 200 Hz. The block had an array of holes spaced 12.7 mm apart in 5 rows and 11 columns (Figure 2e) so that the location and direction of applied force could be changed. All data reported were the maximum magnitude of the resultant force vector across two trials.

### 2.4. Trials and Protocol

To collect grip strength and pinch measurements, participants sat in a chair with their back to the seat, feet flat on the floor, and their elbow slightly extended and slightly abducted. Each participant was asked to grip the dynamometer (Figure 1a) with their whole hand and squeeze as hard as they could for 2–3 s. Additionally, to collect tip and key pinch strength, participants were instructed on the proper placement of the index finger and thumb. Proper hand positioning for tip pinch and key pinch are shown in Figure 1b,c, respectively. All grip and pinch strength measurements were performed 3 times and the average across all trials was reported. Participants were instructed to apply the maximum force possible without causing pain. To collect isolated force, participants were seated in the same testing position for the clinical measurements, with their hand supported on the testing device designed to collect isolated thumb forces (Figure 2). Data collection included isolated thumb forces in two wrist positions: 0° wrist position (Figure 2a,b), which had the palm parallel to the ground; and the 90° wrist position, where the palm was positioned vertically (Figure 2c,d). In each wrist position, two force directions were included: a push, which was described as the thumb pushing the peg “downward” (Figure 2a,c), and a pull, which was described as the thumb pulling the peg in toward the hand (Figure 2b,d). Participants were instructed to execute each push and pull on the peg with their thumb positioned at a comfortable location. The comfortable location was the most comfortable peg location for the individual to contact the peg. All combinations of wrist position and force direction (4 unique testing conditions) were tested twice while exerting maximum force. Additional data collected included the visual analogue scale (VAS) pain score. Participants were asked to report their VAS pain score for their right thumb (for healthy participants) or their surgical thumb (for CMC OA participants). The score represented their self-reported pain level in the 48 h prior to arriving for testing, where a high score represented more pain.

### 2.5. Statistical Analysis

A linear mixed-effects model was used to analyze the isolated thumb force data. Study group (younger healthy males, older healthy males, younger healthy females and older healthy females, pre-surgery, 3-months post-surgery, and 6-months post-surgery), wrist position (0° and 90°), and force direction (push and pull) were labeled as fixed effects. Participants were labeled as random effects to account for the repeated measures within all participants. Interaction terms included group*wrist*direction as well as two-way interactions from the three-way interaction term.

A linear mixed-effects model was conducted to compare grip strength, key pinch, and tip pinch with 0° isolated force data. Study group (younger healthy females, older healthy females, pre-surgery, 3-months post-surgery, and 6-months post-surgery) and measurement type (grip strength, tip pinch, key pinch, 0° isolated pull and 0° isolated push) were included as fixed effects and participants as random effects. The interaction term included measurement type*group.

Statistical analysis was performed using the SAS/STAT software Version 9.4 (SAS Institute Inc., Cary, NC, USA). Denominator degrees-of-freedom were calculated using the Kenward–Roger approximation, and pairwise comparisons were obtained using the Tukey adjustment for all statistical analysis. *p*-values less than 0.05 were considered significant.

## 3. Results

### 3.1. Participants

A total of 52 healthy participants were included in this study: 13 OH females (average age 58.0 ± 9.3 years), 13 YH females (average age 25.8 ± 5.1 years), 13 OH males (average age 60.2 ± 11.4 years), and 13 YH males (average age 28.8 ± 5.6 years).

Thirteen CMC OA females (average age 62.8 ± 8.2 years) were tested. Of these individuals, nine right hands and four left hands underwent the LRTI surgery. Surgery was performed on nine dominant hands and four non-dominant hands. The 13 OA participants were tested prior to surgery (average time before surgery 21.4 ± 31.8 days), 3-months post-surgery (average time after surgery 90.8 ± 8.5 days), and 6-months post-surgery (average time after surgery 182.1 ± 13.9 days). Testing occurred over an 18-month period, which amounted to 39 total testing sessions. Two OA males participated in this study, but due to the small sample size, these data sets were not statistically analyzed or included in this manuscript. Additionally, one OA participant opted to refrain from performing the last pre-surgery trial of 90° force due to pain.

### 3.2. Health Summary

**Figure 3:**
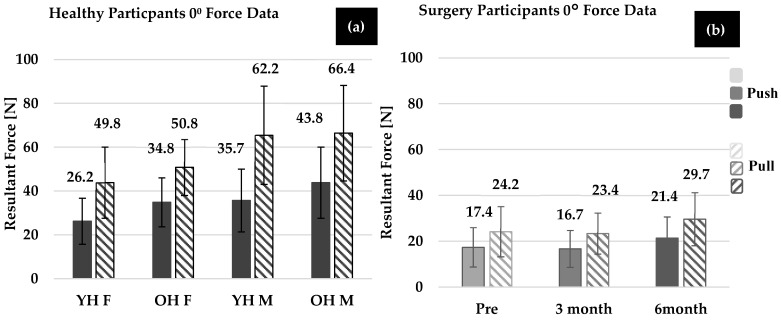
Average maximum force trials for all directions (push and pull) in the 0° location for (**a**) younger and older healthy males and females and (**b**) OA females pre-surgery, 3-months post-surgery, and 6-months post-surgery (n = 13 for each).

**Figure 4:**
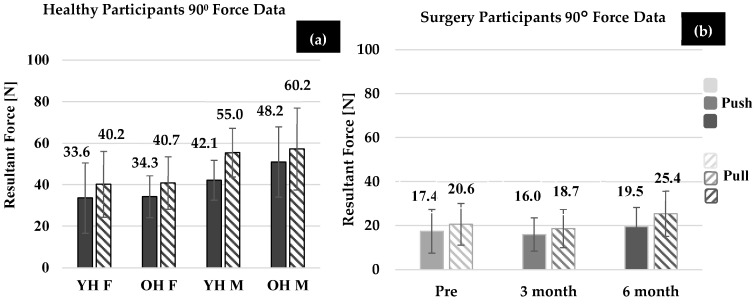
Average maximum force trials for all directions (push and pull) in the 90° location for (**a**) younger and older healthy males and females and (**b**) OA females pre-surgery, 3-months post-surgery, and 6-months post-surgery (n = 13 for each).

Figure 3a and Figure 4a show the average maximum forces for healthy participants in the 0° and 90° wrist positions, respectively. Although several trends were demonstrated, none of the comparisons yielded statistically different results. The trends are reported for completeness. On average, healthy males generated larger thumb force (force direction and wrist position combined) than healthy females in both younger (*p* = 0.992) and older healthy groups (*p* = 0.734). Additionally, OH males and OH females generated more force than YH males and females (males: *p* = 0.880, females: *p* = 0.781), respectively, with both wrist position and force direction combined. In both wrist positions, YH males and OH males generated larger thumb force compared to the YH females and OH females, but this was not found to be significant (*p* > 0.78).

For all healthy groups, the effect of direction (push vs. pull) on force (combining wrist position and group) was found to be significant (*p* < 0.001). The force generated in the 0° thumb pull was statistically larger than the 90° thumb pull (*p* < 0.001), but the 0° thumb push was not statistically different compared to the 90° thumb push (*p* = 0.875, when all groups were combined). On average, OH females exerted more force than YH females in the push direction and pull directions, but comparisons were not statistically significant (push: *p* = 0.158, pull: *p* = 0.510). Additionally, on average, OH males exerted more force than the YH males in the push and pull directions; however, again, comparisons were not statistically significant (push: *p* = 0.118, pull: *p* = 0.219).

The effect of wrist position (when force direction and group were combined) was found to be significant, and more force was generated in the 0° wrist position compared to the 90° wrist position (*p* = 0.007). However, when considering the effect of wrist position within each group (including force direction), only OH females performed statistically greater forces in the 0° compared to the 90° wrist positions (*p* = 0.048).

#### Healthy Clinical Measurement Summary

Clinical measurements, grip strength, tip pinch, and key pinch were collected for all healthy participants (Table 1). No significant differences were found between grip strength, or tip or key pinch. YH males generated more grip and pinch (key and tip) force than OH males, but the difference was not statistically significant (grip, tip, key: *p* > 0.995). OH females showed more grip and tip pinch strength compared to YH females, but the results were also not statistically significant (grip, tip, key: *p* > 0.997).

### 3.3. Surgery Participant Summary

**Figure 5:**
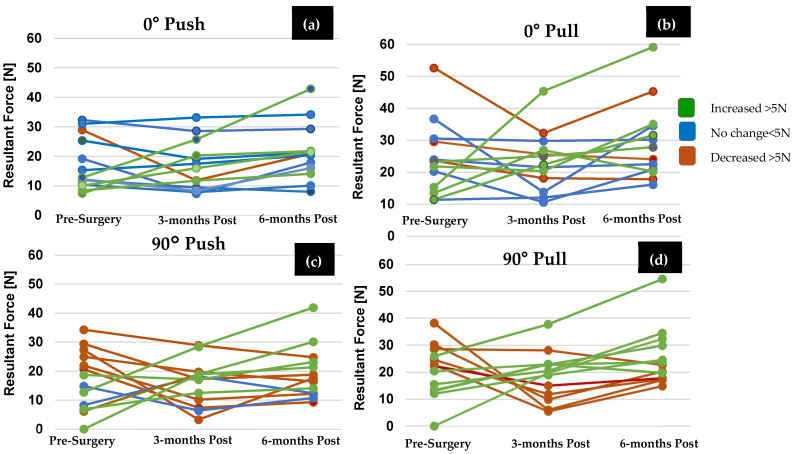
*(***a**) Average maximum force trials for CMC OA females (*n* = 13) in the comfortable location in the (**a**) 0° wrist position (push), (**b**) 0° wrist position (pull), (**c**) 90° wrist position (push), (**d**) 90° wrist position (pull). Participants that showed a decrease (greater than 5 N), increase (greater than 5 N), or no change (within 5 N of pre-surgery force) in thumb force pre– to 6-months post-surgery. One participant declined to execute 90° force trials pre-surgery due to pain.

Individual force data sets of female OA patients were compared at each time point (Figure 3b and Figure 4b). Across all wrist positions and force directions, the average force generated by OA females showed a decreasing trend pre-surgery to 3-months post-surgery (*p* = 0.818), and an improvement at 3-months to 6-months post-surgery (*p* = 0.065) and pre- to 6-months post-surgery (*p* = 0.644), but these trends were not found to be significant. More specifically, on average, OA females increased force at 6-months post-surgery compared to 3-months post-surgery in both the pull and push directions (pull: *p* = 0.528, push: *p* = 0.978). OA females increased force at 6-months post-surgery compared to pre-surgery in the pull (*p* = 0.989) and push (*p* = 0.999) directions. The effect of wrist position on force generation was not found to be significant for OA females at each time point (pre: *p* = 0.975, 3-months: *p* = 0.808, 6-months: *p* = 0.944). Additionally, as seen in Figure 5, groupings of female OA participants were determined to identify an increase (>5 N), a decrease (>5 N), and no change in force abilities (<5 N of pre-surgery force). In most cases, (both the 0° and 90° wrist positions) at least five OA females showed improvement. However, the remainder showed no change or a decrease in force abilities.

#### Surgery Participant Clinical Measurement Summary

**Figure 6:**
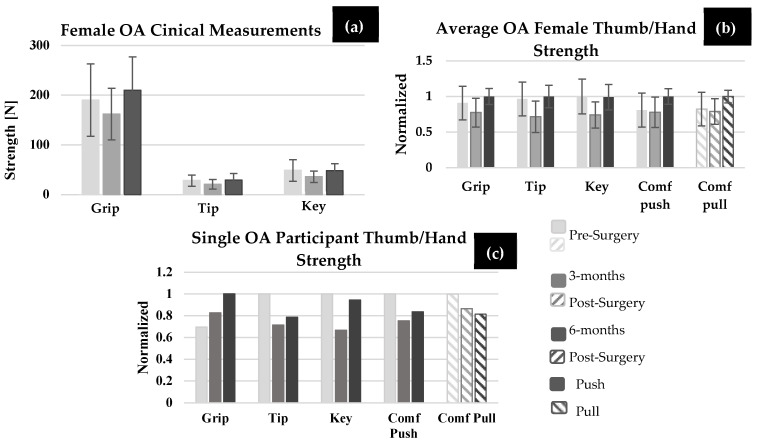
*(***a**) Grip, tip, and key pinch strength for OA females (*n* = 13) pre-surgery, 3-months, and 6-months post-surgery in Newtons. *(***b**) Average grip strength, tip and key pinch strength, 0° push and pull performed for all OA females. (**c**) Grip strength, tip pinch, key pinch, and 0° comfortable push and pull performed by one OA female participant pre-surgery, 3-months, and 6-months post. ((**b**) and (**c**) normalized by the largest value out of all three time points).

Grip strength, tip pinch, and key pinch are shown in Figure 6a for OA females. In the OA female group, five individuals showed an increase in grip, four showed an increase in tip pinch, and five showed an increase in key pinch strength at 6-months post-surgery compared to pre-surgery. No statistical differences were found between any of the time points for any strength measurements (grip strength, tip pinch, and key pinch (*p* > 0.901)), except for grip strength at 6-months post-surgery compared to 3-months post-surgery (*p* = 0.028). For OA participants, key and tip pinch were not significantly different in magnitude for any time point when compared to the 0° comfortable push (*p* > 0.685) or pull force (*p* > 0.999). Pre-surgery (*p* < 0.001) and 3-months post-surgery (*p* < 0.001), OA participants performed significantly less grip strength compared to OH females, but not at 6-months post-surgery (*p* = 0.073). In Figure 6b,c, each clinical measurement and isolated force measurement (0° comfortable thumb push and pull) was normalized by the largest value across the surgical time points. On average, shown in Figure 6b, clinical measurements showed a decrease at 3-months post-surgery compared to pre-surgery, then a slight increase at 6-months compared to pre-surgery (except for key pinch, which decreased by 0.5 N). On average, OA females performed lower push and pull thumb forces pre-surgery compared to tip and key pinch measurement pre-surgery, and therefore larger isolated thumb force improvement was made at 6-months post-surgery, as indicated in Figure 6b. However, not all participants showed the same trend. For example, Figure 6c, which represents one OA female participant’s normalized clinical measurements and isolated thumb force, shows that at 6-months post-surgery, key pinch reached close to that of pre-surgery levels and grip strength increased at each time point post-surgery. However, isolated thumb pull forces showed a reduction in thumb force at 6-months post-surgery compared to pre-surgery. Thus, the recovery trends shown by the clinical measurement tools did not mimic the trends from isolated thumb push and pull forces.

VAS results, shown in Table 2,varied based on each participant. Each participant reported reduced pain at 3-months post-surgery, but this trend did not always continue at the 6-month post-surgery time point. Only two participants reported no pain at 6-months post-surgery; however, six patients reported an increase in pain at 6-months post-surgery compared to 3-months post-surgery, and one participant reported more pain at 6-months post-surgery compared to pre-surgery. Additionally, a larger reduction in pain or 3-months or 6-months post-surgery did not always correlate with an increase in force. For example, participant A1 reported more pain at 6-months post-surgery compared to 3-months post-surgery; however, their isolated thumb force at 6-months post-surgery increased and returned close to its pre-surgery force.

### 3.4. Comparison of Healthy Females and OA Females

Comparisons were also made between the OA and healthy females. At each time point (pre-surgery, 3-months, and 6-months post-surgery), OA female push forces were significantly less than OH females at 3-month post-surgery (*p* = 0.025) but not pre-surgery (*p* = 0.096) or 6-months-post (*p* = 0.247). OA female pull forces were significantly less than the OH females at each time point (pre: *p* = 0.002, 3-month: *p* = 0.001, 6-month: *p* = 0.027).

OA females 3-months post-surgery had significantly different push forces than YH females (*p* < 0.001) 6-months post-surgery. Additionally, at each time point, OA females had significantly less pull force than YH females (all time points: *p* < 0.001).

## 4. Discussion

The goals of this study were to (1) determine and compare thumb force generation in younger and older healthy males and females without CMC OA to those diagnosed with CMC OA pre- and post-LRTI surgery, (2) compare thumb force generation at three time points (pre-surgery, 3-months post-surgery, and 6-months post-surgery) for those who have CMC OA, (3) determine the effect of wrist position on thumb force, and (4) determine the differences between thumb force in the push direction versus a pull direction and compare them to clinical measurements.

### 4.1. Thumb Force Generation between Groups

In this study, comparisons of force generation were made between all groups: YH, OH, and OA (pre-surgery, 3-months post-surgery, and 6-months post-surgery). Isolated thumb force data have not been extensively reported or compared in heathy individuals and those with CMC OA, particularly at multiple points in time related to surgery. The average difference between pre-surgery and 6-months post-surgery force generation varied based on participant; however, only 50% of CMC OA participants showed greater than 5 N of improvement at 6-months post-surgery compared to pre-surgery, and only one participant reached isolated thumb force values of the OH average. Although average force was not restored to the magnitude of healthy females, some CMC OA participants did see improvements in force generation 6-months post-surgery compared to pre-surgery. However, increases in strength levels are not likely to continue after 6-months as patients have ended rehabilitation and research suggests it is unlikely for them to continue their exercises [42].

As expected, on average, healthy (OH and YH) females generated statistically larger isolated thumb forces compared to the OA females pre-surgery. Although not significantly different, the trends showed that OH females generated larger thumb forces than YH females. Additionally, on average, OH male participants generated larger forces than the YH males, although this difference was also not statistically significant. This could be attributed to the fact that the OH individuals had significant work experience in some areas requiring manual labor, whereas the YH group primarily comprised college students with less work experience. Thus, the YH group has less opportunity to develop thumb strength.

### 4.2. Males vs. Females Force Data

Results showed that, on average, healthy males had statistically larger thumb force than healthy females. This was true for both the YH group (males versus females) as well as the OH (males versus females) group, and was consistent with the literature [29,30,43]. It is known that men have more muscle mass than women, especially in their upper body [44,45,46]. Although it was not part of this data collection, historically, some men had occupations that required them to work with their hands, such as in factories, on construction sites, in landscaping, or in carpentry, that could also contribute to the strength differences [47].

### 4.3. Push vs. Pull Force Data

All participants, healthy (OH and YH) and arthritic (pre- and post-surgery), generated larger forces in the pull direction compared to the push direction. In this study, unique postures were used to assess isolated thumb force related to daily movements with comparison of the pull and push directions. The pull position was similar to key pinch in which the thumb adducted toward the other four digits of the hand [48,49]. Muscles that assist in the isolated thumb pull (i.e., CMC flexion) direction are the abductors and flexors surrounding the CMC joint: the adductor pollicis, flexor pollicis longus and brevis, and abductor pollicis longus and brevis [50,51]. While the literature indicates that both the flexor and abductor muscles were active in this motion, the flexors are the stronger of the muscles and highly engaged in the pull forces, while abductor muscles are mainly used as stabilizing muscles [50,51,52]. Therefore, there is a larger functional advantage during thumb pull in comparison to thumb push. Furthermore, the thumb pull direction, or lateral pinch position, is performed in many ADLs such as holding a cup or key. Thumb push is also performed in other ADL tasks such as typing on a keyboard or texting on a phone [53]. Restoring strength to this particular thumb posture is important for individuals who received CMC OA surgery to maintain independent living.

### 4.4. 0°. vs. 90° Wrist Position

Statistical analysis (all groups combined) led to the conclusion that the 0° wrist position produced larger thumb force compared to the 90° wrist position. Studies have also shown that muscles produce their maximum amount of force in their fully lengthened position [54,55,56]. The flexor and abductor pollicis longus are the only two muscles assisting in thumb push and pull that originate on the forearm bones and insert onto the bones of the thumb [57,58,59,60,61]. As the forearm and wrist pronate (from anatomical position), the flexor and abductor pollicis longus are maximally stretched, thus being in a position to produce maximal force. Therefore, in the 0° wrist position (complete pronation of the forearm and wrist from the anatomical position), the two muscles crossing the wrist may assist in generating larger force in the 0° wrist position compared to the 90° wrist position. However, results also showed that when group and direction of force were considered, mixed results were obtained. For example, thumb pull performed in the 0° wrist position generated statistically larger forces compared to thumb pull in the 90° wrist. However, thumb push forces did not show significant differences between the two wrist positions. This difference with regard to wrist position and force direction could have been related to the muscles involved in these activities. The adductor pollicis was reported to be twice as active during lateral pinch (which is similar to isolated thumb pull), making it the dominant muscle along with the flexors, which contribute to the direction of applied force (i.e., pull towards the hand) [50,51]. Additionally, some of the thenar muscles (flexor pollicis brevis and opponens pollicis) assist opposition or thumb pull, which may contribute to the statistically larger forces produced during thumb pull than push.

Comparing the groups, OH females were the only healthy group to generate statistically larger force in the 0° compared to the 90° wrist positions. The literature has shown that ligament laxity around the CMC joint may be a precursor to developing CMC OA. However, force differences between the two wrist positions may not yet be detectible as a loss of function has not become severe. The same significance in force generation between the two wrist positions found in the OH females was not the result for OA females. OA females pre-surgery already have compromised function and this loss of function may have be overshadowed by any force differences related to wrist orientation. These results also revealed that the removal and replacement of the trapezium with ligamentous soft tissue alters the load path and changes the ability to generate force at the level of healthy participants.

### 4.5. Clinical Tools

The outcomes of CMC OA surgery were measured with standard clinical tools including the dynamometer and pinch gauge. On average, patients’ isolated thumb force decreased at 3-months post-surgery, but the findings from the clinical tools did not yield the same results. The challenge with the clinical tools is that the thumb was not isolated, rather other digits were involved in each of the clinical measures. Thus, when measuring pinch strength, the index finger and thumb will exert an equal and opposite force on each side of the pinch gauge, so it is unknown whether the index finger is producing more of the overall force. In this unique study, isolated thumb forces were obtained using a novel measurement device. These data should be used over current methods to monitor the progress of strength abilities after thumb surgery. Many studies in the literature reported that post-surgery thumb strength, using clinical tools, improved compared to pre-surgery, near to that of their healthy contralateral side. However, the data presented here suggested that clinical measures were not a true representation of thumb force abilities [5,6,24,62,63]. Thus, this research provides detailed insight into the thumb force; specifically, how much has been lost, gained, or restored post-surgery compared to pre-surgery and to the healthy population.

## 5. Limitations

Participant activities (e.g., work, leisure) were not known and could have contributed to hand and thumb strength abilities. The occupation of each participant was recorded; however, many of the participants in the OH groups listed their occupation as “retired”. Comparisons of individuals pre- and post-surgery would not be impacted as these are comparisons within an individual. Another limitation is the effort in which participants generated force, as their goal was to generate their maximum amount of force. Thus, it was assumed that each participant was motivated to always produce their maximum level of force. The clinical standard measurement position of the dynamometer and pinch gauge is with the shoulder in neutral position, elbow flexed to 90, and the forearm and wrist in neutral. However, in this study, participants were measured with the elbow extended and shoulder at some degree of abduction, which may have altered the strength levels generated for the maximum grip and pinch strength of each individual. This would likely have little effect on surgical results, as positioning was consistent across surgical timepoints, and would only affect comparisons to the existing literature.

## 6. Conclusions

This study provided comprehensive data sets on the isolated thumb force generation of healthy and arthritic groups post-LRTI surgery. Isolated thumb force has not been extensively reported in the literature, especially for CMC OA individuals and when compared across time points. The data sets reported in this work provide new information to determine if function post-LRTI surgery is at the magnitude of healthy individuals.

This work presented new insights into thumb force generation. A unique method was developed to identify isolated thumb forces in different directions of applied force and wrist positions. Without measurement of isolated thumb force, the effectiveness of surgical intervention, hand therapy, or other treatments cannot be accurately assessed. Current clinical measurement tools have included measurement from other digits of the hand, which do not permit isolated thumb force. Additionally, these tools do not include direction of force or wrist position and their effect on thumb force. Based on the limitations of current thumb force collection and quantification, a different testing procedure is needed. The isolated thumb force protocol in this study is able to detect changes in thumb forces that would otherwise go undetected when utilizing clinical tools. Using these data sets, key differences between the healthy population and those with CMC OA can be used to effectively determine disease onset, disease progression, and force changes pre- and post-surgery. Comparisons at different time points post-surgery and between groups of isolated thumb force data will not be available to clinicians based on current clinical measurements. These data allow surgeons to inform surgical tactics and specified rehabilitation routines, which is an improvement upon current clinical measurement tools. Additionally, these data sets would be beneficial in a clinical setting to identify areas where recovery progress is moving slowly, in which a hand therapist could recommend specific exercises for loss of force in specific thumb postures.

This study laid a foundation using the data collection and analysis approach to conduct future research comparing surgical techniques and therapies post-surgery. Thus, these data sets reporting thumb force in different directions and wrist positions are new findings and important to consider when testing those with CMC OA pre- and post-surgery.

## Figures and Tables

**Table 1:** Average healthy participant grip strength, key pinch, and tip pinch (standard deviation).

	YH Females (*n* = 13)	OH Females (*n* = 13)	YH Males (*n* = 13)	OH Males (*n* = 13)
Grip Strength (N)	264.8 (60.8)	272.6 (41.2)	424.6 (121.6)	419.7 (100)
Tip Pinch (N)	40.2 (10.8)	42.9 (7.8)	61.3 (8.8)	56.5 (7.8)
Key Pinch (N)	75.4 (13.7)	72.0 (12.7)	105.9 (21.6)	104.9 (21.6)

**Table 2:** VAS pain scores for OA participant (*n* = 13) at pre-surgery, and 3-months and 6-months post-surgery.

Participant	Pre	3-Months Post	6-Months Post
A1	25.3	18.1	34.1
A5	87.5	0	22.7
A7	83.7	45.4	35.3
A9	71.9	52.7	57.7
A10	47.0	9.70	1.70
A11	65.7	0	0
A12	67.7	8.60	35.5
A13	23.9	5.40	6.90
A14	19.7	0	0
A15	54.5	31.1	31.8
A16	68.1	0	2.50
A17	38.6	31.8	13.6
A21	71.5	1.75	1.19

## Data Availability

The data presented in this study are available on request from the corresponding author.
